# The impact of antimicrobial stewardship interventions on surgical antibiotic prophylaxis guidelines compliance in a teaching hospital in Ghana

**DOI:** 10.1371/journal.pone.0329541

**Published:** 2025-08-04

**Authors:** Israel Abebrese Sefah, Sarentha Chetty, Peter Yamoah, Varsha Bangalee

**Affiliations:** 1 Discipline of Pharmaceutical Sciences, School of Health Sciences, University of KwaZulu-Natal, Durban, South Africa; 2 Pharmacy Practice Department, School of Pharmacy, University of Health and Allied Sciences, Volta Region, Ghana; Kwame Nkrumah University of Science and Technology Faculty of Pharmacy and Pharmaceutical Sciences, GHANA

## Abstract

**Background:**

Inappropriate use of surgical antibiotic prophylaxis (SAP) to prevent surgical site infections (SSIs) is noted to be a major contributor of antimicrobial resistance (AMR) globally, including Ghana. This study sought to assess the impact of antimicrobial stewardship (AMS) interventions on SAP guideline compliance and other surgical outcomes including SSI and length of hospital stay.

**Method:**

This was a nine-month quasi-experimental (before- and after-intervention) study. Four months’ medical records of 150 obstetric and gynecological surgery patients were collected both before (baseline) and after the implementation of AMS interventions in a teaching hospital in Ghana. The interventions included education on the hospital SAP guidelines and feedback of the baseline survey results to the surgical team. An adapted data collection sheet was used to collect medical records of the included patients. A descriptive analysis was performed, a Pearson’s chi-square test and a two-sample Wilcoxon (Mann-Whitney U) rank-sum test were used to assess the impact of the interventions.

**Results:**

Most (n = 220/300, 73.33%) of the patients were between the ages of 24–44 years, and the commonest surgery was an emergency caesarean section (n = 92/300, 30.67%). After the AMS interventions, there was a significant improvement in guideline compliance due to appropriate antibiotic choice (p = 0.001). duration (p < 0.001), and the volume of consumption of SAP (p < 0.001). There were no significant changes in the timing of administration (p = 0.636), SSI rate (p = 0.054), and the length of hospital stay (p = 0.161).

**Conclusion:**

Our study showed positive impact of the two AMS interventions on SAP guideline compliance based on the choice and duration of prescription, and reduction of the volume of antibiotic utilization, but not on the timing of administration. This did not worsen the SSI rate and patient length of stay. Hospitals in Ghana and beyond are encouraged to optimize SAP use and prevent rising AMR rates by implementing appropriate educational programmes and dissemination of audit findings.

## Introduction

Antimicrobial resistance (AMR) is a global public health problem [1]. It was recognized as a serious global health issue during the 67th and 68th World Health Assemblies in 2014 and 2015 respectively. These culminated in the development of the World Health Organization (WHO) Global Action Plan (GAP) to combat AMR in 2015 and member states were subsequently encouraged to develop their own AMR policy and action plans [2,3]. It has been estimated that the burden of deaths from AMR could rise to 10 million lives each year by 2050 which may lead to a global death toll of one person dying every three seconds if swift action is not taken to slow or combat its occurrence [4]. Globally, 1.14 million deaths, attributed to bacterial AMR was estimated in 2021, with the greatest attributable burden observed in western Sub-Saharan Africa (SSA) [5,6]. This has largely been attributed to the be inappropriate use of antibiotics in humans due to their misuse or overuse accounting for between 20–50 percent of total antibiotic use [7,8] Studies carried out in Ghana have proven the existence of AMR [9,10]. This requires effective Antimicrobial Stewardship (AMS) interventions among other infection prevention and control measures to reduce this [11]. AMS strategies forms part of the five strategic objectives of the Ghana AMR 5-year National Action Plan (NAP) that spanned between 2017 to 2021 [12].

AMS is a coherent set of actions that promote the responsible use of antimicrobials. WHO has identified AMS as one of the three pillars of an integrated approach to health systems strengthening [7]. When this is collaboratively implemented alongside infection prevention and control, and medicine and patient safety, the fight to curb the AMR becomes more effective [11]. AMS has shown the ability to reduce treatment failures, adverse effects and healthcare costs associated with the management of infectious diseases whilst preventing AMR [13]. Interventions implemented may be persuasive (e.g. education, clinical audit with feedback activities to prescribers), structural (e.g. using rapid laboratory testing and therapeutic drug monitoring to improve antibiotic use) or restrictive (e.g. pre-authorization, formulary restrictions and the use of automatic stop orders) [14–16]. Though restrictive interventions have been shown to produce an immediate impact, a combination of persuasive interventions have rather proven to give a more sustainable impact due to the positive behavioral change they have on clinicians’ attitude and practice beyond knowledge influence [14,17,18].

Inappropriate use of surgical antibiotic prophylaxis (SAP), given to prevent surgical site infection (SSI), has been noted to be a major contributor to AMR globally, including low-middle income countries (LMIC) like Ghana [19]. The impact of inappropriate use of SAP may include increased risk of adverse reactions, re-admission rates, prolongation of hospital stays, and increasing healthcare costs as well as a rise in AMR rate. Their appropriate use depends on the selection of the correct choice, dose, timing and duration of administration. About 60% of SSIs can be averted by complying with SAP guidelines [17]. This is important as SSI is considered to be responsible for up to 60% of healthcare-associated infections (HAIs), which occur in about one-third of patients who go through surgery globally [17,20,21]. Another important quality indicator currently being used to assess appropriate antibiotic prescribing is the proportion of ‘Access’, ‘Watch’ and ‘Reserve’ antibiotics prescribed according to the WHO AWaRe classification system, with a recommendation that at least 60% of antibiotics prescribed should be from the ‘Access’ category [22,23]. This system promotes the increased selection of the ‘Access’ group of antibiotics as first line choice for most common infectious disease due to their lower risk of causing AMR [24].

Different AMS interventions such as education, prospective audit of antibiotic prescription and formulary restriction have shown efficacy in improving guideline compliance, reducing SSI rates, duration and cost of SAP [14–16]. Few of such interventional-based studies assessing their impact on SAP have been conducted in Africa and to date none has been conducted in Ghana [25–30]. This study, therefore, sought to provide evidence of the effectiveness of AMS interventions from the Ghanaian context on the appropriate use of SAP and consequently its impact on antibiotic consumption and clinical outcomes of surgical patients; and to assess the influence of sociodemographic and clinical parameters of patients on guideline compliance.

## Methodology

### Study design

This before-and-after interventional study was designed to evaluate the impact of combining education and feedback on a prospective audit of antimicrobial use indicators for surgical patients, provided to clinicians on guideline compliance, volume of antibiotic use and clinical outcomes. The study was conducted in 2 phases comprising of before-intervention phase from October 2023 to January 2024 and the after-intervention phase from May 2024 to July 2024.

### Study setting and population

The study was conducted among patients who underwent surgery in the obstetrics and gynaecology wards of a teaching hospital in the Volta Region of Ghana.

Ghana’s population, according to the 2021 population and housing census report, stood at 30.8 million with the Volta Region being the 7^th^ most populated region [31]. This is one of the 16 administrative regions in the country with Ho as the regional capital and a population of 1,659,040. The hospital is a 306-bed capacity tertiary health facility and is the only teaching hospital in the region serving as the main referral site for advanced medical and surgical care. It has a staff complement of 1,200 and contains 14 wards. It provides primary care and specialized clinical and surgical services (orthopedic, urological, obstetric and gynaecological and general surgeries) to over 20,000 outpatients and inpatients every month who are either inhabitants of Ho Municipality or via referral from cities and towns in the region and beyond [28,32]. This hospital was chosen as a site for this research because it provides both minor and major obstetrics and gynaecology surgeries. Consequently, the hospital attracts a large number of patients seeking such surgical care and thus effective interventions to address the concerns of inappropriate use of SAP may be applicable in several hospital settings in the country and beyond.

### Sample size and sampling technique

An estimated sample size of 150 surgical patients’ electronic medical records were each extracted for both the before and after-arms of the study. Sample size was estimated using Pocock’s formula based on an expected increase in guidelines compliance rate from 14.2% before the intervention to 43.3% after the intervention at a significance level of 5%, a power of 90%, and a 10% adjustment for possible non-compliance in both arms based on a similar study conducted in Nigeria [28,33]. The resulting total sample size of 75 for each phase was doubled to increase the precision of the study. The first 150 medical records of patients who underwent surgical procedure within the study period and were within the inclusion criteria were extracted for both arms of the study.

### Inclusion and exclusion criteria

The electronic medical records of patients who underwent obstetric and gynaecological surgery within the study period, and whose surgical wounds were not contaminated, were included in the study.

Patients who visited the hospital more than once for a similar or different surgical procedure during the study period had their subsequent visit (s) record excluded from the study. Patients whose medical record had missing essential information including the name of the surgical procedure, name, dose, and duration of SAP prescribed, were also excluded.

### Data collection

Data were collected using a checklist designed to extract information from the patient’s electronic medical record from 1^st^ January, 2024 to 31^st^ September, 2024. Patient socio-demographic information extracted included patient identification number, age, gender and national health insurance status (insured or cash payment patient). Clinical information extracted included comorbidity, dates of admission, surgery and discharge, type of wound (clean, clean-contaminated, contaminated), name of surgical procedure, estimated blood loss and SSI diagnosed on admission or within 30 days of discharge. Further information on SAP administered included name of antibiotic prescribed, duration of prophylaxis, timing of administration of first dose before surgical incision commenced, the WHO AWaRE classification of SAP prescribed (either as Access, Watch or Reserve), the length of hospital stay and the volume of antibiotic consumption (oral form, parenteral form and total SAP) measured as Daily Defined Dose (DDD) [34,35].

Patient compliance to hospital SAP guidelines was based on the choice, duration and timing of administration of the first dose of antibiotic for SAP. The guidelines recommended the prescription of a stat dose of parenteral cefuroxime plus metronidazole for elective gynaecological and obstetric surgeries and a 24-hour prescription of parenteral cefuroxime plus metronidazole followed by a 5-day prescription of oral forms of the antibiotics for emergency surgeries.

### Antimicrobial stewardship intervention

A bundle of AMS interventions, including SAP guideline dissemination, education of the surgical team on a variety of topics on AMR and AMS and the dissemination of audit findings of SAP, were tested to assess their impact on the volume and appropriate use of antibiotic, and some clinical outcomes [14].

The SAP guideline/ protocol concerning the choice, duration and timing of administration of antibiotics for the above surgeries was retrieved, verified with the Head of the Department by the principal investigator and details shared with the surgical team during the educational meeting.

The educational meeting material was a PowerPoint presentation of introduction to AMR, definition, principles and interventions of AMS, review of global, regional and local causes of noncompliance to SAP guidelines and comparative analysis of local (hospital) and international SAP guidelines and prescribers’ compliance to SAP baseline survey findings [32,36]. This material was disseminated in a two-hour one-time interactive workshop by the principal investigator on the 15^th^ of May 2024 to a team of obstetrics and gynaecology consultants, specialists, resident medical officers and housemen, anaesthetists, clinical pharmacists and theatre nurses. All participants of the workshop were appropriately registered and a follow-up workshop was arranged for non-participants the following week (22^nd^ May, 2024) to ensure that almost all team members received this education.

A baseline clinical audit of prescribers’ compliance to the choice, duration, and timing of administration of SAP using electronic medical records of patients who underwent surgery in the department from 1^st^ January 2024 to 30^th^ April 2024 was conducted. Findings from this audit were part of the training information disseminated to the surgical team during the one-time interactive workshop. A second audit of electronic medical records of patients who sought for surgical care in this department from 1^st^ June 2024 to 31^st^ September 2024 was also conducted after the intervention implementation to assess the impact of the education and audit with feedback on the stated outcome measures.

### Outcome measures

The impact of the AMS interventions tested were measured using:

1. Antibiotic use indicators: These included determining the proportions of guideline compliance based on the choice, duration and timing of initial dose administration, and of the type of antibiotics used according to the WHO AWaRE classification.
2. Volume of antibiotic consumption before and after the intervention using the WHO Anatomical Therapeutic Chemical (ATC)/ Daily Defined Dose (DDD) [35].
3. Clinical outcomes: These included SSI rate and length of hospital stay.

### Data management and analysis

Data collected using the checklist were entered onto Microsoft Excel version 2016 and imported into STATA version 14 (StataCorp, College Station, TX, USA) for statistical analysis. All categorical variables were presented as frequency and percentages and continuous variables were presented as means and standard deviations. A Chi square test of independence was used to compare the baseline characteristics of patients who attended the hospital before and after the intervention and to assess the differences in the indicators measured (impact of the intervention on guideline compliance) before and after the interventions.

A two-sample Wilcoxon rank-sum (Mann-Whitney U) test was used to evaluate the impact of the interventions on the length of hospital stay, and volume of consumption of SAP (oral, parenteral, and total) expressed as DDD. A multiple logistic regression was used to determine predictors of guideline compliance.

### Ethical consideration

Ethical clearances were sought from the ethical review committees of the University of KwaZulu Natal (BREC/00004236/2022) and the teaching hospital (HTH-REC(35) FC_2023). Study participants’ privacy and confidentiality were safeguarded by anonymizing patient identifiers before accessing the data. Paper-based data collected from the study were kept under lock and key with the principal investigator while the soft data were protected with a password. There were no direct contacts with patients and prescribers during the data collection as data were extracted from patients’ medical records and therefore written informed consents were not sought.

### Results

This results compare the sociodemographic information of patients’ medical records assessed before and after the AMS interventions, and the impact of the interventions on SAP guideline compliance, the quantity of WHO AWaRE class of antibiotics prescribed, the volume of antibiotics consumed and the impact on clinical outcomes.

### Patients’ socio-demographic and clinical characteristics

Out of the total of 300 medical records of patients who underwent obstetric and gynaecological surgery, most (n = 220/300, 73.33%) were between the ages of 24 to 44 years followed by those between the ages of 15 to 25 years (n = 41/300, 13.67). Most (n = 288/300, 96.00%) of the patients were members of the national health insurance. The commonest surgery performed was emergency caesarean sections (n = 92/300, 30.67%) followed by elective caesarean sections (n = 72/300, 24.00%) and most (n = 124/300, 41.47%) of the surgical procedures lasted between 30 to 60 minutes followed by those that lasted between 61–120 minutes (n = 115/300, 38.46%).

There were no significant differences in the age groups (p = 0.465) and the national health insurance status (0.077) of patients who were in the before and after-intervention arms of the study. There was, however, a significant difference between the type of surgery (p < 0.001), the type of surgical wound (p = 0.031) and duration of surgery (p < 0.001) for patients in the two arms of the study (Table 1).

**Table 1:** Comparison of patient socio-demographic and clinical characteristics between before- and after-intervention period.

Variables	Total, n (%)	Intervention Period		*p*-value
		After	Before	
Age groups (years) (n = 300)				0.465
15–25	41 (13.67)	20 (48.78)	21 (51.22)	
26–44	220 (73.33)	115 (52.27)	105 (47.73)	
45–60	26 (8.67)	10 (38.46)	16 (61.54)	
> 60	13 (4.33)	5 (38.46)	8 (61.54)	
Duration of surgery (minutes) (n = 299)				<0.001*
< 30	29 (9.70)	6 (20.69)	23 (79.31)	
30–60	124 (41.47)	82 (66.13)	42 (33.87)	
61–120	115 (38.46)	50 (43.48)	65 (56.52)	
121–180	24 (8.03)	7 (29.17)	17 (70.83)	
181–240	5 (1.67)	2 (40.00)	3 (60.00)	
> 240	2 (0.67)	2 (100.00)	0 (0.00)	
NHIS status (n = 300)				0.077
Insured	288 (96.00)	147 (51.04)	141 (48.96)	
Not insured	12 (4.00)	3 (25.00)	9 (75.00)	
Wound type (n = 300)				0.031*
Clean	72 (24.00)	44 (61.11)	28 (38.89)	
Clean-contaminated	228 (76.00)	106 (46.49)	122 (53.51)	
Surgery type (n = 300)				<0.001*
Elective caesarean section	72 (24.00)	44 (61.11)	28 (38.89)	
Emergency caesarean section	92 (30.67)	63 (68.48)	29 (31.52)	
Gynecological laparotomy	4 (1.33)	0 (0.00)	4 (100.00)	
Myomectomy	43 (14.33)	15 (34.88)	28 (65.12)	
Salpingectomy	7 (2.33)	1 (14.29)	6 (85.71)	
Exploratory laparotomy	14 (4.67)	6 (42.86)	8 (57.14)	
Total abdominal hysterectomy	25 (8.33)	6 (24.00)	19 (76.00)	
Bilateral tubal ligation	2 (0.67)	1 (50.00)	1 (50.00)	
Cervical cerclage	10 (3.33)	0 (0.00)	10 (100.00)	
Others	31 (10.33)	14 (45.16)	17 (54.84)	

P-values (*) are those less than 0.05.

### Impact of AMS interventions on guideline compliance and patient clinical outcomes

The implementation of a combination of education of the surgical team on hospital SAP guidelines coupled with the provision of feedback of baseline audit findings of prescribers’ compliance to the guidelines had significant impact on compliance. This included compliance to guidelines based on choice (p = 0.001) and duration SAP prescription (p < 0.001).

The interventions above had no impact compliance based on the timing of administration of SAP (p = 0.636), overall guideline compliance (p = 0.169), WHO AWaRE class of antibiotic prescribed (p = 0.996), the rate of SSI (p = 0.054) and the length of hospital stay (p = 0.088) of the patients (Table 2).

**Table 2:** The impact of AMS intervention on guideline compliance and clinical outcomes.

Variables	Total, n (%)	Intervention Period		*p*-value
		After	Before	
Compliance based on SAP choice (n = 297)				0.001*
Yes	274 (92.26)	144 (52.55)	130 (47.45)	
No	23 (7.74)	4 (17.39)	19 (82.61)	
Compliance based on SAP duration (n = 297)				<0.001*
Yes	241 (81.14)	135 (56.02)	106 (43.98)	
No	56 (18.86)	14 (25.00)	42 (75.00)	
Compliance based on SAP timing of administration (n = 268)				0.636
Yes	244 (91.04)	130 (53.28)	114 (46.72)	
No	24 (8.96)	14 (58.33)	10 (41.67)	
Overall guidelines compliance (n = 297)				0.169
Yes	223 (75.08)	106 (47.53)	117 (52.47)	
No	74 (24.92)	42 (56.76)	32 (43.24)	
WHO AWaRE Classification (n = 297)				0.996
Access + Watch	295 (99.33)	147 (49.83)	148 (50.17)	
Access	2 (0.67)	1 (50.00)	1 (50.00)	
Surgical site infection diagnosis (n = 300)				0.054
Yes	10 (3.33)	2 (20.00)	8 (80.00)	
No	290 (96.67)	148 (51.03)	142 (48.97)	
Length of hospital stay (days) (n = 300)				0.088
< 7	214 (71.33)	115 (53.74)	99 (46.26)	
7–13	56 (18.67)	26 (46.43)	30 (53.57)	
14–20	16 (5.33)	4 (25.00)	12 (75.00)	
21–27	11 (3.67)	3 (27.27)	8 (72.73)	
> 27	3 (1.00)	2 (66.67)	1 (33.33)	

P-values with asterisk (*) symbol are those less than 0.05.

### Predictors of overall guideline compliance of SAP prescription

Overall SAP guideline compliance based on choice, duration and timing of their administration was reduced by 95% (aOR=0.043, CI 0.019–0.102, p < 0.001) when SAP dosing was beyond 24 hours compared when given within 24 hours. Also, the occurrence of SSI was associated with 89% decrease in overall guideline compliance (aOR=0.105, CI 0.014–0.789, p = 0.047). Lastly, guideline compliance was also reduced by 90% (aOR=0.093, CI 0.019–0.469, p = 0.004) when the patient had no active national health insurance status (Table 3).

**Table 3:** Predictors of overall guideline compliance.

Variables	Adjusted Odds Ratio	Confidence interval (95%)	*p*-value
**Age group years (n = 300)**			0.278
15–25 (r)	1.000		
26–44	1.306	0.468–3.647	0.609
45–60	3.729	0.723–19.251	0.116
> 60	3.373	0.468–24.297	0.227
**SAP Dosing (n = 300)**			0.483
24 hrs. dosing (r)	1.000		
Single dosing	1.165	0.398–3.409	0.780
> 24hr dosing	0.043	0.019–0.102	<0.001*
**WHO AWaRE Classification (n = 274))**			0.864
Access (r)	1.000		
Access + Watch	2.449	0.072–83.163	0.618
**Surgical Site Infection (n = 10)**			0.047*
Yes	0.105	0.014–0.789	0.029*
No (r)	1.000		
**National Health Insurance Status (n = 300)**			0.003*
Insured (r)	1.000		
Not insured	0.093	0.019–0.469	0.004*
**Type of Surgery (n = 300)**			0.671
Bilateral Tubal Ligation (r)	1.000		
Cervical cerclage	1.000		
Elective caesarean section	0.440	0.021–9.104	0.595
Emergency caesarean section	1.078	0.051–22.758	0.961
Exploratory laparotomy	0.580	0.020–16.443	0.750
Gynecological laparotomy	1.000		
Myomectomy	0.621	0.029–13.229	0.760
Salpingectomy	0.515	0.012–21.360	0.727
Total abdominal hysterectomy	0.130	0.006–2.992	0.203
Others	0.604	0.026–14.021	0.753

r refers to reference sub-variable used for the regression analysis; P-values (*) with asterisk symbol are those less than 0.05.

### Impact of individual guideline compliance parameters

Guideline compliance based on the choice of SAP prescribed was associated with the type of WHO AWaRE class of antibiotics (p = 0.025) but had no association with SSI occurrence (p = 0.786) and the length of hospitalization of the patient (p = 0.428).

Guideline compliance based on the duration of SAP prescribed was associated with occurrence of SSI (p = 0.010) but had no association with the type of WHO AWaRE class of antibiotic prescribed (p = 0.259) and the length of hospitalization of the patient (p = 0.470).

Guideline compliance based on the timing of administration of SAP was associated with the length of hospitalization of the patient (p < 0.001) but had no association with the type of WHO AWaRE class of antibiotic prescribed (p = 0.655) and the occurrence of SSI (p = 0.437) (Table 4).

**Table 4:** Impact of Individual Guideline Compliance Parameters on Selected Characteristics.

Variables	Choice Compliance			Duration Compliance			Timing Compliance		
	Yes n (%)	No n (%)	p-value	Yes n (%)	No n (%)	p-value	Yes n (%)	No n (%)	p-value
**WHO AWaRE Classification (n = 274)**			0.025*			0.259			0.655
Access	1 (50.00)	1 (50.00)		1 (50.00)	1 (50.00)		2 (100.00)	0 (0.00)	
Access + Watch	273 (92.54)	22 (7.54)		240 (81.36)	55 (18.64)		240 (90.91)	24 (9.09)	
**Surgical Site Infection (n = 10)**			0.786			0.010*			0.437
Yes	9 (90.00)	265 (92.33)		5 (50.00)	5 (50.00)		238 (90.84)	24 (9.16)	
No (r)	1 (10.00)	22 (7.67)		236 (82.23)	51 (17.77)		6 (100.00)	0 (0.00)	
**Length of hospitalization (days) (n = 274)**			0.428			0.470			<0.001*
< 7	195 (92.42)	16 (7.58)		168 (79.62)	43 (20.38)		175 (90.21)	19 (9.79)	
7–13	51 (51.00)	5 (8.93)		50 (89.29)	6 (10.71)		48 (94.12)	3 (5.88)	
14–20	15 (93.75)	1 (6.25)		12 (75.00)	4 (25.00)		12 100.00)	0 (0.00)	
21–27	11 (100.00)	0 (0.00)		9 (81.82)	2 (18.18)		9 (100.00)	0 (0.00)	
> 27	2 (66.67)	1 (33.33)		2 (66.67)	1 (33.33)		0 (0.00)	2 (100.00)	

### Impact of AMS interventions on the volume of SAP utilization, duration of hospitalization and SAP prescribed

The AMS interventions implemented had a significant impact on reducing mean volume of intravenous SAP (p = 0.021), oral SAP (p < 0.001), total SAP (combination of intravenous and oral dosage forms) (p < 0.001) expressed as DDD and the mean duration of SAP prescribed (p < 0.001). The interventions however had no impact on the reduction of the length of hospitalization of the patients (p = 0.161) (Table 5).

**Table 5:** The impact of AMS intervention on SAP utilization, length of hospitalization, and duration of antibiotic use.

Variables	Intervention Period		*p*-value
	After	Before	
Intravenous SAP used (DDD) (mean ±SD)	1.30 ± 0.05	1.57 ± 0.07	0.021*
Oral SAP use (DDD) (mean ±SD)	3.59 ± 0.60	14.59 ± 0.65	< 0.001*
Total SAP used (DDD) (mean ±SD)	4.89 ± 0.61	16.17 ± 0.67	< 0.001*
Length of hospitalization (mean ±SD)	6.08 ± 0.39	7.25 ± 0.48	0.161
Duration of SAP prescription (mean ±SD)	2.39 ± 0.22	6.64 ± 0.23	< 0.001*

P-values (*) less than 0.05 after the application of the two-sample Wilcoxon rank-sum (Mann-Whitney) test.

### The types of SAP prescribed

**Fig 1:**
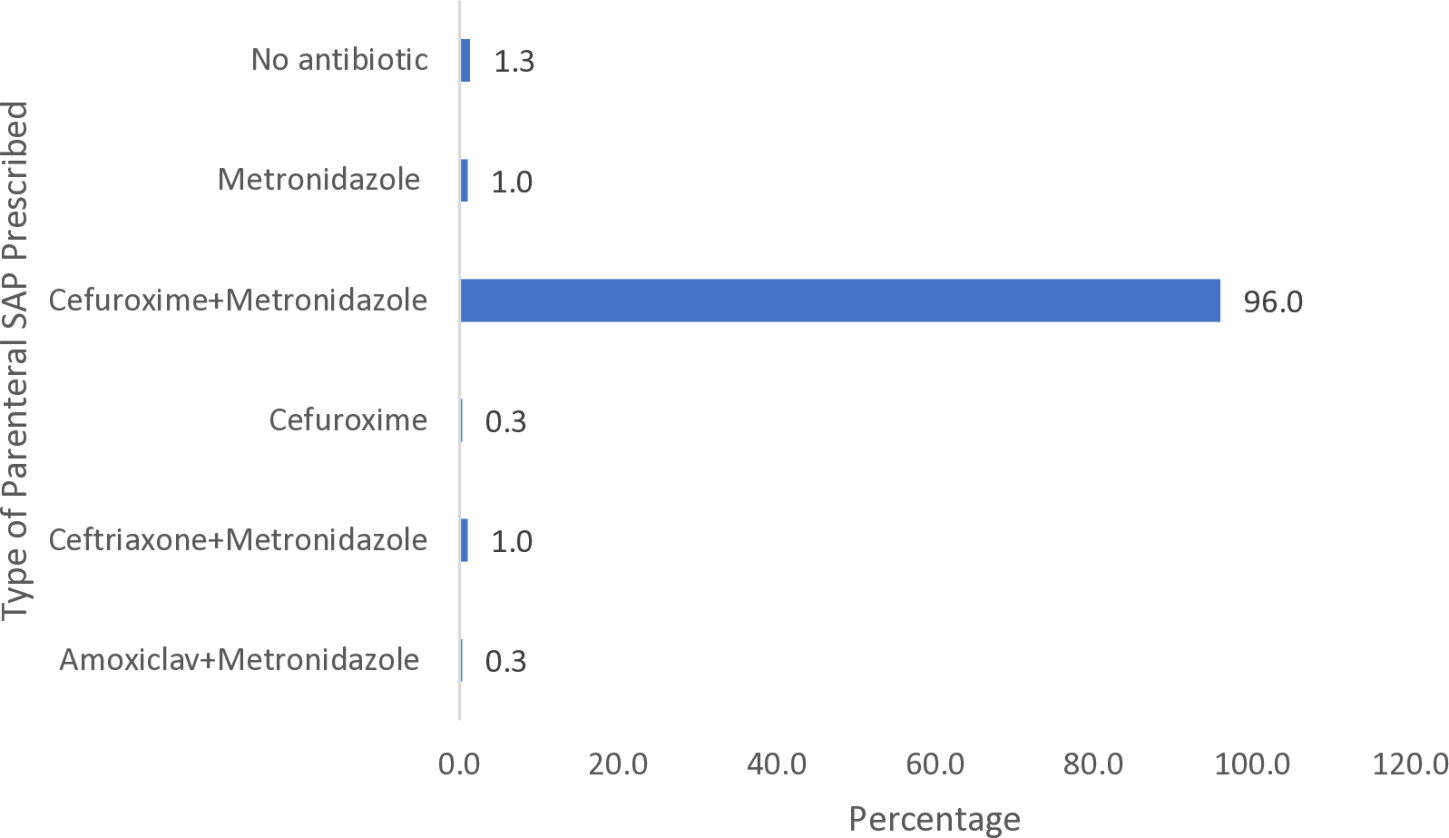
Percentage of Parenteral SAP Prescribed (n = 300).

The commonest type of SAP prescribed in the parenteral dosage form was cefuroxime plus metronidazole (96.0%). Only 1.3% of the patients had no parenteral antibiotic prescribed for them as prophylaxis (Fig 1).

**Fig 2:**
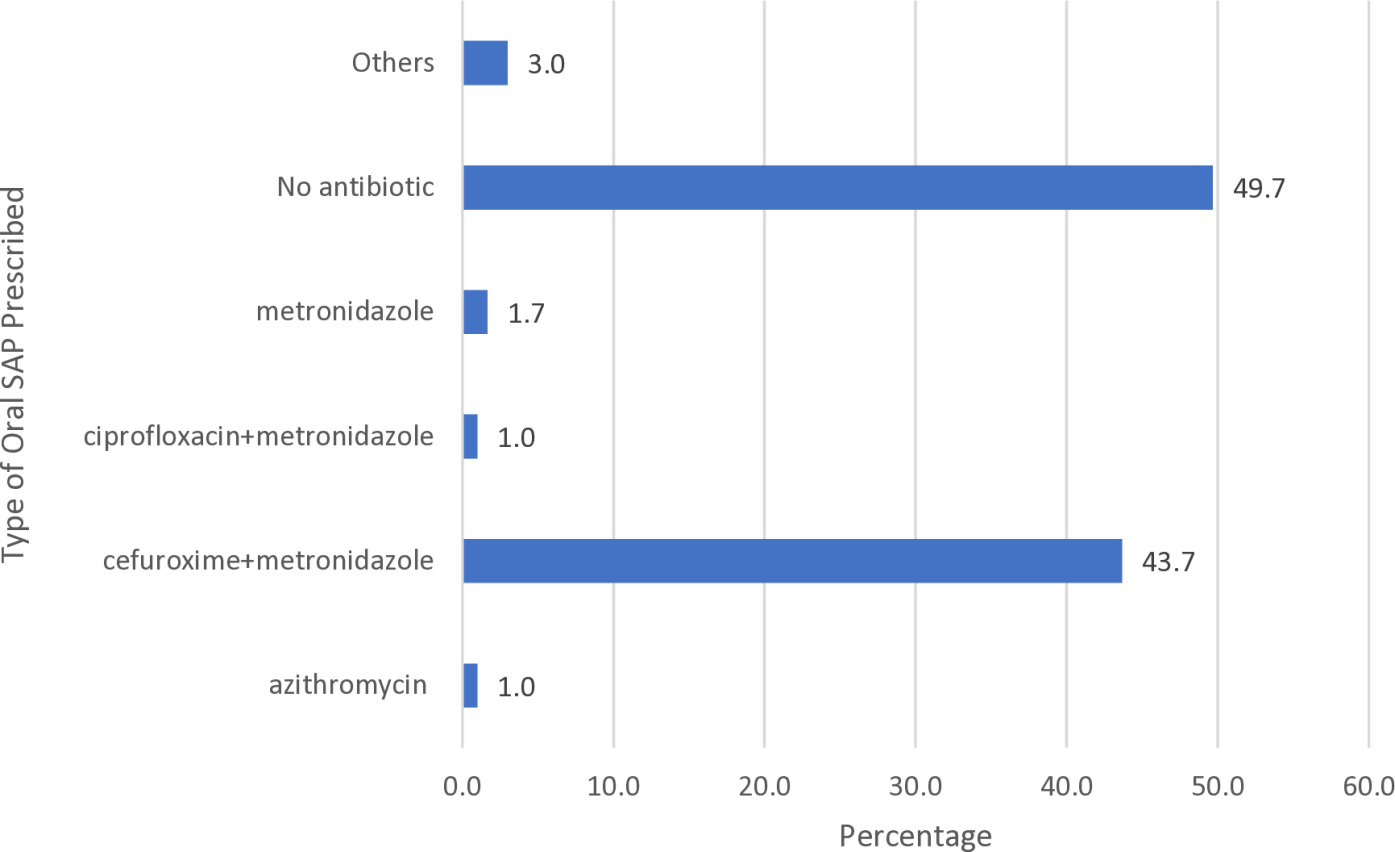
Percentage of Oral Prescribed (n = 300).

The commonest type of SAP prescribed in the oral dosage form for the patients was cefuroxime plus metronidazole (43.7%). Almost half (49.7%) of patients were not prescribed with oral SAP (Fig 2).

## Discussion

To the best of our knowledge this is the first study of this type to be conducted in a Teaching Hospital in Ghana to assess the impact of AMS interventions on SAP guideline compliance, and its impact on antibiotic consumption and clinical outcomes of surgical patients.

The study showed that a combination of surgical team education on SAP guidelines with dissemination of baseline audit findings to prescribers resulted in a significant improvement in guideline compliance on the choice of SAP and their duration of prescription but had no impact on the timing of initial dose administration. These findings are consistent with several studies performed elsewhere that showed a significant decrease in SAP duration and an increase in the appropriate selection of antibiotics per guidelines after implementing AMS interventions [30,37–39]. Though our study showed no impact of AMS interventions on the timing of the initial dose of SAP administration per guidelines, some earlier studies have shown a positive impact on this indicator [25,28,30,39]. Others still showed an impact on the choice, duration and timing of SAP administration [30,37,39]. The above evidence demonstrates the effectiveness of AMS interventions on the improvement of SAP guidelines compliance which has positive consequences on patient outcomes and measures to address misuse and overuse of SAP to curb rising AMR rates.

While some of these quasi-experimental studies were conducted among patients who underwent urological, orthopedic, cardiothoracic, and gastrointestinal surgeries [37–40], many others were done among patients who underwent obstetric and gynecological surgeries similar to this study [25,28,41,42]. This is encouraging as it demonstrates that the correct application of AMS interventions to improve SAP guidelines compliance is not restricted to specific surgical specialties.

AMS intervention could be persuasive, structural or restrictive in its application [14,17,27]. In our study, we employed a combination of two persuasive interventions that have been shown by previous studies to produce a more sustainable impact on prescribing behavior [17,18]. This is in contrast to restrictive interventions which produce immediate but non-sustainable impact on behavioral change [14].

The AMS interventions also had a positive impact on the volume of antibiotic consumption by the reduction of intravenous SAP given before and after surgery and oral SAP given mostly after intravenous therapy to patients who are able to tolerate oral medications. This has potential benefits such as the reduction of possible adverse drugs reaction due overuse of antibiotics, reduction in healthcare cost, and reduction in the risk of increased AMR rates due to increased selective pressure on micro-organisms due to misuse and overuse of SAP [17,36,43].

The implementation of a combination of education on SAP guidelines coupled with an audit with subsequent feedback of findings to clinicians had no significant impact on the length of hospitalization of patients, surgical site infections. This demonstrates that guideline compliance, which included a reduction in the duration of SAP, did not lead to a worsening of these clinical outcomes. There have been concerns about the possible negative impact of the reduction in the duration of SAP on patients’ outcome such as the increase in surgical site infections rate in hospitals in developing countries due to poor infection prevention and control practices, overcrowding, and patient malnutrition (36). There was also no impact on the type of WHO AWaRE class of antibiotics prescribed which may only change based on the decision to include more Access antibiotics, which has lower risk of promoting resistance development compared to those in the Watch and Reserve class, in the hospital guidelines [22,23].

Overall guidelines compliance (combination of appropriate choice, duration and timing of administration) was significantly associated with SAP dosing was beyond 24 hours compared to when given within 24 hours, the occurrence of a SSI after a surgery and when patient had no national health insurance. These factors contributed to the to the non-significant improvement in the overall SAP guideline compliance in this teaching hospital. Appropriate AMS programme must be instigated to further investigate these factors to help in the design of an effective strategy to achieve overall guideline compliance. The above findings are in contrast with other similar studies that achieved optimal improvement in overall guidelines [30,37,38], while it agrees with others that reported non-significant improvement [26,28,44].

The commonest SAP regimen prescribed for both parenteral and oral administration was a combination of cefuroxime and metronidazole. This regimen differs from SAP antibiotics used in similar studies carried out in the obstetric and gynecological department, where options include only cefuroxime, a combination of ampicillin and metronidazole, only co-amoxiclav, ampicillin combined cloxacillin and metronidazole, and only cefazolin [25,28,29,41,42]. Some notable international guidelines including the WHO antibiotic guideline recommends cefazolin which belongs to the WHO Access class of antibiotics and poses minimal risk to AMR development [24]. The 2022 National Caeserean Section Guidelines for the Society of Gynaecologists and Obstetricians of Ghana (SOGOG) recommends the prescription of co-amoxiclav or cefuroxime as SAP for caesarian section surgeries [45].

The study had some limitations including the non-randomization of the patients; the limited sample size; the conduct of the study in a single study site and the collection of data from only obstetric and gynaecological ward, and the limited sociodemographic and clinical characteristics data that were collected that could have confounded the impact of the interventions on guidelines compliance. These possible confounders have the potential to limit the internal and external validity of the findings. The study was also limited by the possible effect of a change in hospital policy on antibiotic usage or Hawthorne effect from change of behavior of prescribers on causality of the intervention due to their non-assessment in this survey. We believe that the findings of this study in Ghana will provide useful information for researchers in this area to develop a more robust design to further investigate the above findings while providing vital evidence for policy makers and practitioners to develop effective AMS to improve SAP guidelines compliance and its intended consequences on patient outcomes.

## Conclusion and recommendation

The combination of surgical team education and dissemination of audit findings to the team led to an improvement in SAP guidelines compliance due to appropriate choice and the duration of antibiotic prescription but not on the timing of initial dose administration. The impact of the interventions on choice of SAP did not affect the WHO AWaRE class of antibiotic prescribed. The AMS interventions also resulted in a significant reduction in the volume of utilization of both parenteral and oral antibiotics used for SAP. The improved compliance and reduced utilization of antibiotics did not result in the worsening of clinical outcomes including surgical site infection rate and the length of hospitalization of the patients. Guideline compliance was reduced by the use of SAP beyond 24 hours, occurrence of surgical site infection and the patient’s possession of a national health insurance.

Hospitals in Ghana and beyond are encouraged to optimize SAP use as part of measures to avoid worse surgical outcomes and prevent rising AMR rates due to overuse and misuse of antibiotics by implementing educational programmes and dissemination of audit findings to members of the healthcare team.
